# Inflammation and Cardiovascular Risk: A Systematic Review of High-Sensitivity CRP as a Prognostic Indicator

**DOI:** 10.7759/cureus.103348

**Published:** 2026-02-10

**Authors:** Syeda Umm E Abeeha Zaidi, Ahmad Mohammad, Ishtiaq Ahmad, Shivam Singla, Bhavna Singla, Sunita Kumawat, Sajid Abbas

**Affiliations:** 1 Internal Medicine, Islamabad Medical & Dental College, Islamabad, PAK; 2 Internal Medicine, Hurley Medical Center, Flint, USA; 3 Internal Medicine, University of Iowa Hospitals and Clinics, Iowa City, USA; 4 Internal Medicine, TidalHealth Peninsula Regional, Salisbury, USA; 5 Internal Medicine, Erie County Medical Center, Buffalo, USA; 6 Internal Medicine, St. Francis Medical Center, Lynwood, USA; 7 Internal Medicine, Holy Family Hospital, Rawalpindi, Rawalpindi, PAK

**Keywords:** cardiovascular risk, hs-crp, inflammation, major adverse cardiovascular events, prognostic biomarker, residual risk, risk stratification, systemic inflammation, type 2 diabetes, vascular mortality

## Abstract

This systematic review evaluates the prognostic role of high-sensitivity CRP (hs-CRP) in predicting cardiovascular outcomes among individuals with type 2 diabetes. Across prospective cohorts spanning varied populations and risk strata, elevated hs-CRP levels consistently signaled heightened susceptibility to major adverse cardiovascular events, vascular mortality, and, in some cases, microvascular complications. Importantly, hs-CRP retained predictive relevance even after controlling for traditional risk indicators such as glycemic status, lipid profiles, and blood pressure, underscoring inflammation as an independent driver of cardiovascular risk in diabetes. Divergent findings in a subset of studies, where hs-CRP more strongly predicted mortality than nonfatal ischemic events, suggest that the biomarker may reflect systemic inflammatory vulnerability and impaired recovery capacity rather than solely plaque burden. The collective evidence supports hs-CRP as a clinically meaningful, biologically plausible indicator of residual risk not captured by conventional models. Incorporating hs-CRP into cardiovascular risk stratification frameworks for diabetic patients may enhance individualized prevention strategies and justify future research on targeted anti-inflammatory interventions. Overall, this review highlights inflammation as a measurable and potentially modifiable determinant of cardiovascular outcomes in diabetes, reinforcing the clinical value of hs-CRP beyond its observational association.

## Introduction and background

Cardiovascular disease (CVD) remains the leading cause of morbidity and mortality among individuals with diabetes mellitus, representing nearly two-thirds of all deaths in this population. Diabetes accelerates atherosclerosis through chronic hyperglycemia, dyslipidemia, oxidative stress, and endothelial dysfunction, creating a pro-atherogenic milieu that persists even with modern therapeutic regimens [1]. Despite advances in glucose-lowering medications, lipid control, antiplatelet therapy, and blood pressure management, residual cardiovascular risk in diabetics remains substantial. This gap highlights the need for reliable biomarkers capable of identifying high-risk individuals earlier, refining risk stratification, and guiding preventive strategies [2,3].

High-sensitivity CRP (hs-CRP) has emerged as one of the most widely studied inflammatory biomarkers in cardiovascular medicine. Produced predominantly by the liver in response to IL-6 and other cytokines, hs-CRP reflects low-grade inflammation involved in plaque formation, progression, and instability [4,5]. Unlike traditional risk markers that depict metabolic or structural abnormalities, hs-CRP captures the inflammatory component of atherosclerosis, a dimension increasingly recognized as central to cardiovascular events. Its assay sensitivity allows detection of subtle elevations that precede clinical outcomes, making it a candidate predictor rather than merely a responder to vascular injury [6].

Diabetes further amplifies inflammation through mechanisms such as advanced glycation end-product accumulation, adipocyte-mediated cytokine release, and chronic endothelial irritation. Consequently, hs-CRP levels tend to be higher in diabetic patients than in nondiabetic counterparts, and this elevation may correlate with microvascular and macrovascular complications [7]. Observational cohorts have shown that higher hs-CRP levels in diabetes are linked with increased risk of myocardial infarction, stroke, coronary artery disease progression, and cardiovascular mortality, independent of traditional risk factors. Importantly, hs-CRP may complement conventional risk stratification tools by improving prognostic discrimination and identifying patients who would benefit from intensified preventive interventions [8].

Despite accumulating evidence, uncertainty persists regarding the consistency, magnitude, and independence of hs-CRP’s prognostic value across diverse diabetic populations and clinical contexts. Variability in sample size, baseline cardiovascular risk profiles, assay thresholds, adjustment for confounders, and outcome definitions complicates interpretation. Additionally, whether hs-CRP meaningfully adds predictive value beyond established markers, such as HbA1c, LDL cholesterol, and blood pressure, is still debated. Clarifying these associations is crucial for determining whether hs-CRP should move from being a research biomarker to a practical clinical tool integrated into cardiovascular risk assessment algorithms for diabetes management. Therefore, the objective of this study is to systematically evaluate the prognostic role of hs-CRP in predicting cardiovascular events among diabetic patients, synthesizing evidence from prospective cohort studies to determine its predictive strength, independence, and potential clinical utility.

## Review

Materials and methods

Study Design and Framework

This research was conducted as a systematic review of cohort-based prognostic evidence evaluating the relationship between hs-CRP and cardiovascular outcomes in individuals with type 2 diabetes. The review followed the Preferred Reporting Items for Systematic reviews and Meta-Analyses (PRISMA) guidelines [9] to ensure transparency, reproducibility, and methodological rigor. A review protocol was developed a priori but was not prospectively registered in PROSPERO or any other international database. The search was restricted to full-text, peer-reviewed articles published in English, with no limitations on year or country of publication. Only original cohort analyses were considered primary evidence; previously published reviews or meta-analyses were excluded and used only for contextual interpretation.

Eligibility Criteria (PICO Model)

Eligibility criteria were defined using a prespecified PICO framework [10]. Studies were included if they enrolled adults with type 2 diabetes, irrespective of disease duration, geographic region, ethnicity, or baseline cardiovascular risk. The exposure of interest was baseline hs-CRP (or CRP measured using high-sensitivity assays), reported either continuously or in categorical groupings. Eligible comparators included lower versus higher hs-CRP strata, reference quartiles, or per-unit increment models. Outcomes required cardiovascular morbidity or mortality, including coronary heart disease events, major adverse cardiovascular events, cardiovascular death, stroke, or composite endpoints, with secondary outcomes encompassing all-cause mortality and microvascular complications. Only prospective cohort studies and prognostic analyses derived from randomized trial populations were considered, whereas animal studies, cross-sectional designs, case-control studies, and mechanistic investigations were excluded.

Search Strategy and Study Selection

Electronic searches were performed in PubMed, Google Scholar, Scopus, and Web of Science using combinations of keywords including “hs-CRP”, “CRP”, “diabetes”, “cardiovascular risk”, “major adverse cardiovascular events”, and “prognosis”. Reference lists of key articles and related reviews were scanned to identify additional eligible studies. Titles and abstracts were screened for relevance, followed by full-text assessment according to PICO criteria. Eight studies met inclusion thresholds, representing evidence across European, Asian, and North American cohorts.

Data Extraction and Management

Key study characteristics were extracted using a structured template designed for prognostic research. Extracted variables included author and year, cohort design, sample size, population features, hs-CRP measurement approach, follow-up duration, cardiovascular outcomes evaluated, statistical modeling strategy, adjusted hazard/relative risk estimates, and covariates included in multivariable models. To ensure analytical consistency, whenever hs-CRP was presented categorically, effect sizes were recorded comparing the highest versus the lowest exposure groups; when reported continuously, per-unit changes were retained.

Quality Appraisal and Risk of Bias Assessment

Risk of bias for each included study was appraised using the Quality In Prognosis Studies (QUIPS) tool [11], which evaluates bias across domains including study participation, attrition, exposure measurement, outcome definition, confounding, and statistical analysis. Each study was independently judged and assigned a concise overall rating of low or moderate risk. Most included cohorts exhibited moderate risk due to potential residual confounding or incomplete reporting of attrition, whereas one post-trial prognostic dataset demonstrated lower risk due to rigorous event adjudication and consistent follow-up.

Synthesis of Evidence

Evidence synthesis was descriptive and comparative. Given heterogeneity in populations, hs-CRP categorization, analytic models, and outcome definitions, quantitative pooling (meta-analysis) was not attempted. Instead, results were narratively synthesized to evaluate consistency in direction and magnitude of prognostic associations across settings. This approach allowed triangulation of findings from large post-acute coronary syndrome (ACS) datasets, population-based cohorts, and studies without CVD, enhancing clinical interpretability.

Ethics and Reporting Considerations

As this review synthesized already published data, no ethical approval was required. The review was conducted and reported in accordance with PRISMA, with predefined selection criteria, transparent data extraction, structured quality assessment, and justification for narrative synthesis.

Results

Study Selection Process

**Figure 1:**
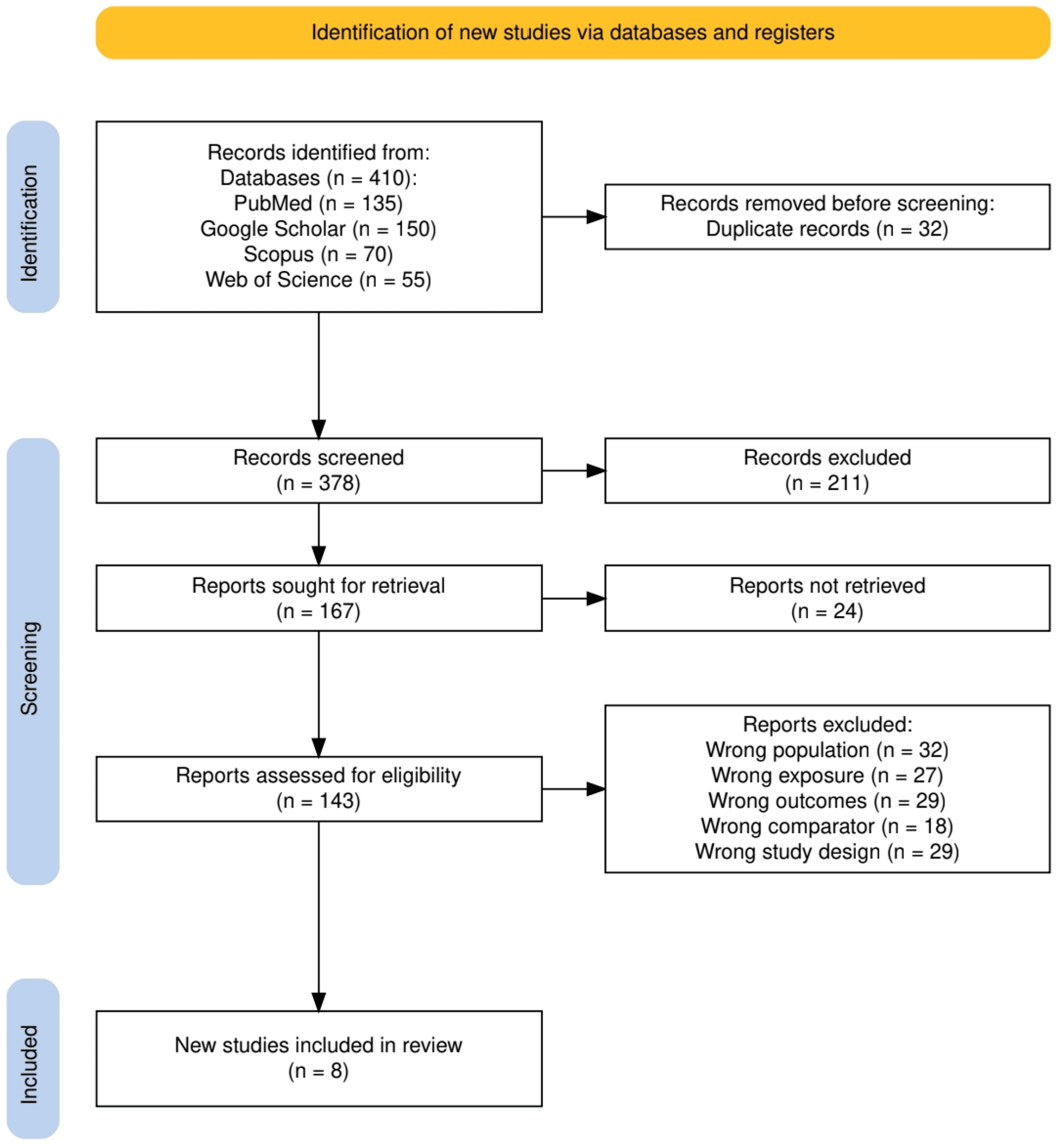
PRISMA flowchart showing the study selection process PRISMA, Preferred Reporting Items for Systematic reviews and Meta-Analyses

A total of 410 records were identified through database searches, from which 32 duplicates were removed prior to screening. After title and abstract review, 378 records were screened, and 211 were excluded. Full texts of 167 reports were sought, of which 24 could not be retrieved. The remaining 143 full-text papers were assessed for eligibility, leading to 135 exclusions based on predefined criteria, including wrong population, exposure, outcomes, comparator, or study design. Ultimately, eight studies met the inclusion thresholds and were retained for synthesis, as illustrated in Figure 1.

Characteristics of the Selected Studies

The eight studies included in this review (Table 1) collectively represent a diverse and clinically relevant spectrum of type 2 diabetes populations across geographic settings, risk profiles, and outcome definitions. Sample sizes ranged widely, from smaller mechanistic cohorts of approximately 350 participants to larger trial-embedded analyses exceeding 5,000 individuals, enhancing both depth and external validity. Follow-up duration varied from just over two years to nearly eight years, allowing assessment of both short-term and long-term prognostic effects. Most cohorts consisted of middle-aged to older adults, with baseline CVD either excluded to capture incident risk or deliberately included to evaluate recurrence risk. Despite variability in hs-CRP categorization, ranging from quartile-based comparisons to absolute thresholds (>3 mg/L) and continuous modelling, the studies consistently utilized validated high-sensitivity assays. Cardiovascular outcomes also differed across cohorts, encompassing composite major adverse cardiovascular events, coronary deaths, stroke, and all-cause mortality, while one study extended applicability to microvascular disease. Importantly, all studies applied multivariable statistical adjustment, underscoring attempts to isolate the independent contribution of hs-CRP to vascular risk. Overall, the characteristics summarized in Table 1 reflect a mature body of prospective evidence with methodological consistency in biomarker assessment and outcome ascertainment, but also sufficient heterogeneity to allow exploration of differential risk patterns across patient subgroups.

**Table 1:** Summary of included studies evaluating the association between hs-CRP levels and cardiovascular outcomes ACS, acute coronary syndrome; BP, blood pressure; CABG, coronary artery bypass grafting; CAD, coronary artery disease; CHD, coronary heart disease; CV, cardiovascular; CVD, cardiovascular disease; hs-CRP, high-sensitivity CRP; LDL-C, low-density lipoprotein cholesterol; MI, myocardial infarction; RCT, randomized controlled trial

Study	Study design	Sample size (n)	Population characteristics (age, diabetes type, and baseline CVD status)	Exposure measurement (hs-CRP assay, cutoff/categorization)	Follow-up duration	Outcomes assessed	Statistical model used	Effect size (HR/RR/OR with CI)	Adjustment variables included
Schulze et al. (2004) [12]	Prospective cohort	746	American men aged 46-81 years with type 2 diabetes; no baseline CVD	CRP categorized into quartiles	Mean 5 years	Composite CV events (MI, CABG/angioplasty, and stroke)	Multivariate Cox regression	RR across quartiles: 1.00, 1.51, 2.52, 2.62; p-trend = 0.011	Age, BMI, smoking, alcohol use, physical activity, family history, hypertension, cholesterol history, aspirin use, fibrinogen, creatinine, HbA1c, non-HDL cholesterol
Soinio et al. (2006) [13]	Prospective cohort	1,045	Adults aged 45-64 years with type 2 diabetes; subgroup without prior MI analyzed separately	hs-CRP categorized at >3 mg/L vs. ≤3 mg/L	7 years	CHD mortality and incident (fatal/nonfatal) MI	Cox regression analysis	HR 1.72 for CHD death after adjustment; among MI-free subjects, HR 1.84 for CHD death	Adjusted for multiple confounders, including cardiovascular risk factors
Matsumoto et al. (2003) [14]	Prospective cohort	350	Japanese adults with type 2 diabetes; baseline CVD history partially accounted for	hs-CRP analyzed as continuous (per SD) and tertiles	1-7 years (mean 4.5 years)	All-cause mortality and nonfatal CAD or stroke	Multivariable models / Cox regression	Per SD increase: HR 1.30 (95% CI 1.04-1.67); highest vs. lowest tertile: HR 2.00 (95% CI 1.03-3.85)	Age, systolic BP, smoking, insulin resistance index, prior CVD
Bruno et al. (2009) [15]	Population-based prospective cohort	2,381	Adults with type 2 diabetes; subgroup analyses included normoalbuminuric patients and those without baseline CVD	CRP categorized at >3 mg/L vs. ≤3 mg/L	Median 5.4 years	All-cause mortality and cardiovascular mortality	Multivariate Cox proportional hazards modeling	HR 1.51 (95% CI 1.18-1.92) for all-cause mortality; HR 1.44 (95% CI 0.99-2.08) for CV mortality	Albumin excretion rate, HbA1c, blood pressure, baseline CVD status, standard cardiovascular risk factors
Lee et al. (2011) [16]	Retrospective cohort with prospective follow-up	1,558	Korean adults with type 2 diabetes; no baseline CVD	hs-CRP categorized: <0.08 mg/dL vs. >0.21 mg/dL (lowest vs. highest)	Mean 55.5 months	Major adverse cardiovascular events	Cox proportional hazards model	HR 1.77 (95% CI 1.16-2.71) for the highest vs. the lowest hs-CRP category	Age, physical activity, smoking, and diabetes duration
Aryan et al. (2018) [17]	Population-based prospective cohort	1,301	Adults with type 2 diabetes; individuals with hs-CRP >20 mg/L excluded	Baseline hs-CRP measured continuously (per SD increment)	Mean 7.5 years	Coronary heart disease events; diabetic retinopathy, neuropathy, and kidney disease	Cox proportional hazards models	HR per SD increase: 1.028 (95% CI 1.024-1.032) for CHD; 1.025 (95% CI 1.021-1.029) for neuropathy; 1.037 (95% CI 1.030-1.043) for retinopathy; 1.035 (95% CI 1.027-1.043) for nephropathy	Adjusted for traditional vascular risk factors; reclassification metrics also assessed
Sharif et al. (2021) [18]	Prospective cohort (SMART study)	1,679	High-risk type 2 diabetes patients with or without prior vascular disease	hs-CRP analyzed as a log-transformed continuous variable	Median 7.8 years (IQR 4.2-11.1)	Composite CV events (MI, stroke, and vascular mortality) and all-cause mortality	Cox proportional hazards models	HR per 1-unit log(hs-CRP): 1.21 (95% CI 1.01-1.46) for vascular mortality; 1.26 (95% CI 1.10-1.45) for all-cause mortality	Age, sex, BMI, smoking, alcohol use, non-HDL cholesterol, microalbuminuria
Hwang et al. (2017) [19]	Post hoc prognostic analysis of an RCT cohort (EXAMINE trial)	5,380	Type 2 diabetes with recent ACS	hs-CRP categories: <1, 1-3, and >3 mg/L; also dichotomized ≤3 vs. >3 combined with LDL-C	30 months	Major adverse cardiovascular events (CV death, nonfatal MI, and nonfatal stroke)	Cox proportional hazards models	HR 1.42 (95% CI 1.13-1.78; p = 0.002) for hs-CRP >3 mg/L vs. <1 mg/L	Adjusted for relevant confounders, including LDL-C

Risk of Bias Assessment

The risk of bias across the included studies was systematically assessed using standardized methodological criteria, focusing on key domains such as selection bias, performance bias, detection bias, attrition bias, and reporting bias. Overall, the majority of studies demonstrated a low to moderate risk of bias, with clear descriptions of study design, outcome assessment, and follow-up. However, some studies exhibited potential concerns related to incomplete outcome data and lack of blinding, which may influence the internal validity of their findings. A detailed summary of the risk of bias assessment for each included study is presented in Table 2.

**Table 2:** Risk of bias assessment of studies evaluating hs-CRP and cardiovascular risk hs-CRP, high-sensitivity CRP

Study	Risk of bias summary	Final judgment
Schulze et al. (2004) [12]	Well-defined prospective cohort with validated outcomes and extensive adjustment; however, moderate potential for residual confounding and incomplete attrition reporting.	Moderate
Soinio et al. (2006) [13]	Large cohort with hard outcomes and multivariable adjustment; details on follow-up losses are limited, and residual confounding is likely.	Moderate
Matsumoto et al. (2003) [14]	hs-CRP appropriately measured and outcomes clinically robust; smaller sample size and variable follow-up introduce a moderate risk of bias.	Moderate
Bruno et al. (2009) [15]	Population-based cohort with reliable mortality endpoints and appropriate covariate control; modest missing sampling data and limited individual follow-up information.	Moderate
Lee et al. (2011) [16]	Clear hs-CRP exposure classification and Cox modeling; hospital-based design and limited confounder control increase the risk of bias.	Moderate
Aryan et al. (2018) [17]	Large diabetic cohort with continuous hs-CRP modeling and improvement metrics; attrition description is limited, and residual confounding cannot be excluded.	Moderate
Sharif et al. (2021) [18]	Well-defined high-risk cohort, robust event verification, and adequate adjustment; referral-based population limits generalizability.	Moderate
Hwang et al. (2017) [19]	Post-trial cohort with high internal validity, adjudicated outcomes, and strong analytical adjustment; lowest risk among included studies.	Low

Discussion

Across diverse diabetic populations, the reviewed evidence consistently demonstrates that elevated hs-CRP is a clinically meaningful predictor of cardiovascular risk. Schulze et al. (2004) [12] showed a clear risk gradient, with cardiovascular event rates rising steadily across CRP quartiles, suggesting that inflammation may accelerate vascular instability. This pattern was echoed by Soinio et al. (2006) [13], where patients with hs-CRP >3 mg/L experienced nearly double the risk of coronary death, even in those without prior myocardial infarction, indicating that hs-CRP may capture latent vulnerability before overt disease manifests. Matsumoto et al. (2003) [14] strengthened this interpretation by showing that inflammation predicts both fatal and nonfatal events and that risk doubles in the highest tertile of hs-CRP. Meanwhile, Bruno et al. (2009) [15] demonstrated that CRP predicts not only cardiovascular but also all-cause mortality, expanding the prognostic relevance beyond traditional endpoints. Notably, Lee et al. (2011) [16] and Hwang et al. (2017) [19] confirmed this association in Asian and post-ACS cohorts, respectively, supporting external validity across ethnic and clinical strata. Sharif et al. (2021) [18] added nuance by showing that hs-CRP relates more strongly to mortality than to nonfatal ischemic events, suggesting that the biomarker may better reflect systemic frailty and inflammatory vulnerability rather than the burden of occlusive disease alone. Finally, Aryan et al. (2018) [17] broadened the scope by linking hs-CRP to both macrovascular and microvascular complications, implying that inflammation is not specific to coronary events but may reflect general vascular injury pathways. Collectively, these findings imply that hs-CRP is not merely an associative marker but a biological signal of pathogenic processes intrinsically linked to diabetic vascular risk.

Traditional cardiovascular risk models in diabetes emphasize lipid status, glycemic control, blood pressure, and renal markers, yet residual risk persists despite optimal treatment. The reviewed studies highlight inflammation indexed by hs-CRP as a mechanistically and clinically relevant variable rarely incorporated into risk stratification algorithms. Hwang et al. (2017) [19] elegantly illustrated this gap by demonstrating that hs-CRP identifies risk independent of and additive to LDL-cholesterol reduction, indicating that lipid-targeted therapy does not mitigate the inflammatory component of vascular disease. Similarly, Schulze et al. (2004) [12] and Soinio et al. (2006) [13] found hs-CRP predictive even after controlling for HbA1c, adiposity, and blood pressure, reinforcing the idea that current clinical frameworks underestimate the inflammatory drivers of diabetic atherosclerosis. Aryan et al. (2018) [17] further suggested that inflammation spans microvascular and macrovascular domains, meaning traditional cardiometabolic markers only capture a fraction of risk biology. Sharif et al. (2021) [18] advanced this concept by revealing that hs-CRP was more strongly associated with mortality than with nonfatal ischemic events, implying that chronic inflammation may influence vulnerability to collapse rather than isolated plaque rupture. Thus, the collective literature positions hs-CRP as a window into residual risk biology, specifically inflammatory dysregulation, that remains invisible to cholesterol and glycemic indices. Integrating hs-CRP into diabetes risk profiling may therefore identify patients who would benefit from anti-inflammatory strategies, beyond conventional metabolic and lipid-lowering interventions [20].

A dominant theme emerging from the included studies is that hs-CRP retains predictive value even after accounting for established vascular determinants such as glycemic control, lipid levels, and blood pressure, highlighting the biomarker’s unique contribution to risk biology. Schulze et al. (2004) [12] showed that CRP quartiles predicted cardiovascular events independent of HbA1c, non-HDL cholesterol, and hypertension, implying that inflammation may precede or amplify the effects of metabolic injury. Soinio et al. (2006) [13] similarly demonstrated that hs-CRP remained associated with coronary mortality after adjusting for lipid status, smoking, and blood pressure, reinforcing that inflammation represents a parallel pathogenic axis rather than a downstream effect. Hwang et al. (2017) [19] extended this concept by showing hs-CRP was additive to LDL-lowering effects after ACS, suggesting that even aggressive statin therapy fails to neutralize inflammation-driven risk. Collectively, these observations imply that hs-CRP does not simply mirror metabolic dysfunction but captures a mechanistically distinct and clinically relevant risk dimension, supporting the argument that targeting inflammation may represent a necessary therapeutic complement to glucose and lipid management in diabetes [21].

The observation that hs-CRP predicts vascular mortality more strongly than nonfatal events, as seen in Sharif et al. (2021) [18], offers conceptual insight into the biology that hs-CRP reflects. One plausible explanation is that elevated hs-CRP represents systemic inflammatory vulnerability, or frailty, involving endothelial instability, autonomic imbalance, and immune dysregulation, making fatal collapse more likely than isolated ischemic episodes. Meanwhile, nonfatal myocardial infarction or stroke may depend more heavily on structural atherosclerotic burden rather than inflammatory activation alone, which could explain why some cohorts showed weaker associations for incident MI than for cardiovascular death. Matsumoto et al. (2003) [14] and Bruno et al. (2009) [15] support this distinction by reporting stronger predictive effects for mortality outcomes. This divergence suggests that hs-CRP may function less as a marker of plaque volume and more as a signal of plaque instability, impaired recovery capacity, or systemic inflammatory aging. Understanding this difference may help refine clinical use-positioning hs-CRP as a tool for mortality risk stratification rather than for event prediction alone [22].

The systematic consistency with which hs-CRP improves risk prediction raises important translational questions about whether it should eventually be incorporated into clinical scoring models such as ASCVD, UKPDS, or SCORE2-Diabetes [23]. Aryan et al. (2018) [17] demonstrated improved discrimination and net reclassification for both microvascular and macrovascular endpoints when hs-CRP was added to traditional risk factors, providing empirical justification for further methodological exploration. Hwang et al. (2017) [19] similarly showed that hs-CRP refined prognostication even in lipid-managed post-ACS patients, illustrating its capacity to detect residual risk not captured by LDL-driven frameworks. However, it must be emphasized that no prospective clinical trials have yet evaluated hs-CRP-guided treatment strategies in type 2 diabetes, and therefore any potential implementation pathway remains hypothetical. The concept of inflammation-based stratification tiers, such as prioritizing more intensive preventive measures or exploring anti-inflammatory approaches currently being tested in nondiabetic populations [24], should be viewed strictly as part of a future research agenda rather than as a clinical recommendation. Incorporating hs-CRP into diabetes risk algorithms may ultimately improve individualized prevention, but this requires prospective validation before informing guideline-based practice.

The observed prognostic role of hs-CRP is supported by a robust biological framework linking inflammation to vascular injury in diabetes. Chronic hyperglycemia induces oxidative stress, AGE-mediated endothelial damage, and adipocyte dysfunction, stimulating IL-6 release, which drives hepatic synthesis of CRP; thus, elevated hs-CRP reflects upstream inflammatory signaling rather than passive bystander activity [8,25]. Matsumoto et al. (2003) [14] reinforced this connection by showing that inflammation and insulin resistance exert additive effects on mortality and cardiovascular events, suggesting shared yet partially independent metabolic pathways. Mechanistically, inflammation promotes endothelial dysfunction, reduces nitric oxide bioavailability, and accelerates monocyte adhesion, setting the stage for plaque formation and instability. Moreover, hs-CRP itself may propagate vascular injury by enhancing LDL uptake by macrophages, impairing endothelial repair, and affecting fibrinolytic balance. The stronger relationships observed for mortality in studies such as Bruno et al. (2009) [15] and Sharif et al. (2021) [18] imply that systemic inflammatory vulnerability increases not only plaque rupture risk but also resilience failure after ischemic injury. Thus, hs-CRP serves as an integrative biomarker capturing immune activation, metabolic stress, vascular fragility, and diminished repair capacity, making its predictive capacity physiological rather than merely statistical.

Confidence in these conclusions is strengthened by several methodological advantages across studies: large sample sizes (e.g., [15,19]), multinational representation spanning European, Asian, and North American populations, and diverse risk spectrums ranging from asymptomatic diabetics [12] to post-ACS cohorts [19]. Consistent adjustment for traditional risk variables gives credibility to the independent effect of hs-CRP. The inclusion of microvascular outcomes, as shown by Aryan et al. (2018) [17], broadens the relevance beyond coronary endpoints. However, residual confounding remains a universal limitation, as not all inflammatory drivers were accounted for, and observational designs preclude causality. Heterogeneity in hs-CRP reporting (continuous vs. categorical thresholds) complicates cross-study comparison, and few studies explored treatment interactions, leaving it unclear whether modifying hs-CRP improves outcomes. Attrition and endpoint ascertainment were not uniformly reported across cohorts, contributing to moderate risk of bias ratings. Nonetheless, the convergence of findings across settings and designs suggests the signal is biologically meaningful, albeit requiring cautious clinical interpretation.

To contextualize these findings, several methodological limitations should be acknowledged. First, although a narrative synthesis was employed due to considerable heterogeneity in study populations, hs-CRP categorizations, analytical models, and outcome definitions, this approach inherently carries a risk of subjective interpretation and selective emphasis despite the use of structured extraction and comparative assessment frameworks. Second, the inability to retrieve 24 of the 167 full-text articles identified during screening raises the possibility of selective nonretrieval bias, as unavailable studies may have differed systematically from those included. These factors may influence the completeness and balance of the evidence base and should be considered when interpreting the overall strength and generalizability of the conclusions.

Several important gaps emerge from the evidence and create opportunities for impactful research. First, given the independent and additive prognostic role of hs-CRP demonstrated in cohorts such as Hwang et al. (2017) [19], clinical trials should evaluate whether anti-inflammatory therapeutics, such as IL-1β inhibitors, colchicine, or structured lifestyle anti-inflammatory interventions, can reduce event rates specifically in high-hs-CRP diabetic populations. Second, findings from Sharif et al. (2021) [18] suggest that hs-CRP may be a stronger marker of mortality than nonfatal ischemic events, raising questions about whether it reflects systemic frailty or impaired compensatory reserve; future studies should explore differential risk pathways. Third, research should stratify diabetics by phenotypes such as visceral adiposity-driven inflammation, insulin-resistant metabolic syndrome, or autoimmune-linked inflammatory diabetes, potentially refining biomarker use toward precision cardiometabolic medicine. Finally, predictive models incorporating hs-CRP warrant validation against decision-making endpoints, such as treatment escalation or intervention allocation, to clarify whether hs-CRP should transition from an observational marker to an actionable clinical variable. Addressing these gaps may enable hs-CRP to evolve from a prognostic signal to a mechanistically targeted therapeutic guide.

## Conclusions

This systematic review highlights that hs-CRP is a robust and independent predictor of cardiovascular outcomes in individuals with type 2 diabetes, retaining its prognostic value even after adjustment for glycemic control, lipid status, and blood pressure. Consistent associations across diverse populations and clinical settings reinforce the concept that inflammation represents a distinct and clinically relevant pathway driving residual vascular risk beyond traditional metabolic factors. Importantly, elevated hs-CRP not only signals susceptibility to major adverse cardiovascular events but also appears to reflect broader vascular vulnerability, including mortality and microvascular complications. The significance of this review lies in demonstrating that inflammation is measurable, clinically meaningful, and potentially actionable. These findings support considering hs-CRP within cardiovascular risk stratification frameworks for diabetes and provide a strong rationale for future research into targeted anti-inflammatory strategies. Addressing this inflammatory dimension of risk may meaningfully improve precision prevention and help bridge the persistent gap between predicted and observed cardiovascular outcomes in diabetic populations.
